# Iron overload in *Plasmodium berghei*-infected placenta as a pathogenesis mechanism of fetal death

**DOI:** 10.3389/fphar.2014.00155

**Published:** 2014-07-01

**Authors:** Carlos Penha-Gonçalves, Raffaella Gozzelino, Luciana V. de Moraes

**Affiliations:** Instituto Gulbenkian de Ciência, OeirasPortugal

**Keywords:** pregnancy malaria, heme, iron, fetal death, trophoblast

## Abstract

*Plasmodium* infection during gestation may lead to severe clinical manifestations including abortion, stillbirth, intrauterine growth retardation, and low birth weight. Mechanisms underlying such poor pregnancy outcomes are still unclear. In the animal model of severe placental malaria (PM), *in utero* fetal death frequently occurs and mothers often succumb to infection before or immediately after delivery. *Plasmodium berghei*-infected erythrocytes (IEs) continuously accumulate in the placenta, where they are then phagocytosed by fetal-derived placental cells, namely trophoblasts. Inside the phagosomes, disruption of IEs leads to the release of non-hemoglobin bound heme, which is subsequently catabolized by heme oxygenase-1 into carbon monoxide, biliverdin, and labile iron. Fine-tuned regulatory mechanisms operate to maintain iron homeostasis, preventing the deleterious effect of iron-induced oxidative stress. Our preliminary results demonstrate that iron overload in trophoblasts of *P. berghei*-infected placenta is associated with fetal death. Placentas which supported normally developing embryos showed no iron accumulation within the trophoblasts. Placentas from dead fetuses showed massive iron accumulation, which was associated with parasitic burden. Here we present preliminary data suggesting that disruption of iron homeostasis in trophoblasts during the course of PM is a consequence of heme accumulation after intense IE engulfment. We propose that iron overload in placenta is a pathogenic component of PM, contributing to fetal death. The mechanism through which it operates still needs to be elucidated.

## INTRODUCTION

Malaria is an infectious disease that affects millions of individuals every year and remains one of the major causes of morbidity and mortality worldwide (WHO | World Malaria Report, 2013). The disease is transmitted by *Anopheles* mosquito carrying *Plasmodium* parasites. Once injected in the blood stream, *Plasmodium* replicate and mature in the liver (liver stage) before infecting erythrocytes (blood stage; Schofield and Grau, 2005). The blood stage of infection is known to be associated to clinical manifestations of the disease (Mackintosh et al., 2004; Haldar et al., 2007). *Plasmodium* parasites proliferate inside red blood cells; within these erythrocytes circulate freely in the bloodstream and are under risk of removal by the spleen – the organ responsible for eliminating old and modified erythrocytes (Janicik et al., 1978). To avoid this host defense mechanism, intracellular parasites export to the erythrocyte membrane molecules that interact with receptors on the vascular endothelium (Krücken et al., 2005), enabling adherence/sequestration of the infected erythrocyte (IE) in the microvasculature of specific organs such as lung, heart, and brain (Pongponratn et al., 1991). IE sequestration is considered one of the major factors in triggering organ inflammation, leading to severe clinical forms of disease such as cerebral malaria (Carter et al., 2005; Ponsford et al., 2012). It has recently been shown that proliferation and maturation of mutant parasites lacking ability to adhere to host endothelium is reduced in mice when compared to *wild-type* parasites (Fonager et al., 2012) strengthening the hypothesis that adherence impacts *Plasmodium* proliferation during the blood stage of infection.

In 2007 over 125 million women living in areas with *Plasmodium falciparum* and or *P. vivax* transmission became pregnant and at risk of developing malaria (Dellicour et al., 2010). Pregnant women bitten by infected mosquitoes can develop placental malaria (PM; known also as pregnancy malaria) – a disease characterized by adverse pregnancy outcomes such as abortion, stillbirth, premature delivery and low birth weight babies, which in turn increases infant morbidity. These clinical features can be recapitulated in a BALB/c mouse model of infection at mid-stage pregnancy (Neres et al., 2008) and are associated to accumulation of *Plasmodium* IEs in the placenta (Fried and Duffy, 1996).

The placenta is a favored niche for IE sequestration and there are two different explanations for this phenomenon. Placental cells, specifically trophoblasts which are epithelial cells of fetal origin, express chondroitin sulfate A (CSA) – a sulfated glycosaminoglycan – on their surface (Duffy and Fried, 1999). This offers a binding site for VAR2CSA, a protein encoded for the human malaria parasite *P. falciparum,* exposed on the membrane of IEs (Salanti et al., 2003, 2004; Duffy et al., 2006; Srivastava et al., 2010). Physical CSA–VAR2CSA interaction allows the adhesion of IEs to placental tissue. Microcirculatory dynamics of the placenta is another important factor in IE sequestration. We recently described that the speed of maternal blood circulation in the mouse placenta is heterogeneous: there are areas of high, moderate, and low blood flow (de Moraes et al., 2013). In placentas infected with *P. berghei* – a rodent parasite that lacks the VAR2CSA molecule (Jemmely et al., 2010) – accumulation of IEs is increased in low maternal blood flow regions (de Moraes et al., 2013). This highlights the relevance of placental tissue configuration in promoting sequestration of *Plasmodium* IEs. We hypothesized that IE sequestration may occur through specific interaction of IEs with the trophoblast membrane and is favored by IE arrest in maternal regions characterized of low blood flow.

Placental IE sequestration leads to a local inflammatory response characterized by monocyte infiltration (Abrams et al., 2003). Trophoblasts are also capable of phagocytosing IEs (de Moraes et al., 2013) yeast and bacteria (Amarante-Paffaro et al., 2004) in an attempt to eliminate invading microbes. Trophoblast responses to infection may be deleterious to the developing fetus. Despite many evidences that link PM to placental inflammation, mechanisms underlying poor pregnancy outcomes are still unclear.

Our preliminary data in a mouse model of PM show an association between iron overload in trophoblast of *P. berghei*-infected placentas and fetal death. Iron accumulation was observed in 43% of placentas from dead fetuses and was associated to the dose of injected IEs. Iron deposits were never detected in placentas from live embryos. Our preliminary results also show downregulation of mRNA expression of the heme exporter feline leukemia virus C receptor 1a (FLVCR1a) in infected placentas suggesting a role for this molecule in dysregulation of iron homeostasis in PM. Here, we discuss how iron is regulated during pregnancy and develop a hypothesis to explain infection-induced iron overload in trophoblasts. This may help explain the poor pregnancy outcomes brought about by *Plasmodium* infections during gestation.

## IRON AND PREGNANCY

### MATERNAL IRON HOMEOSTASIS

Iron is essential for all living organisms. It is important in a variety of biological functions involving reduction and oxidation reactions which are crucial for cell survival and proliferation (Crichton and Charloteaux-Wauters, 1987). Iron can exchange electrons with a number of different substrates, necessitating tight control of the reactivity of this metal (Hentze et al., 2004). Control is provided by cellular and systemic mechanisms that have been evolved to maintain iron homeostasis and prevent its participation in the Fenton chemistry (Fenton, 1894). Disruption of iron homeostasis leads to production of highly reactive hydroxyl radicals, the cytotoxic effect of which is associated with tissue iron overload, organ dysfunction and disease severity, recently demonstrated in the case of severe forms of malaria (Gozzelino et al., 2012).

Dietary iron is absorbed by the duodenum, transported into the cytosol by the divalent metal transporter-1 (DMT1; Fleming et al., 1997; Gunshin et al., 1997; Veuthy and Wessling-Resnick, 2014) and then either stored within multimeric subunits of ferritin (Harrison and Arosio, 1996) or released into the circulation by the iron exporter ferroportin (Donovan et al., 2000; McKie et al., 2000). Extracellular iron is bound to transferrin (Tf) and delivered to cells by interaction with Tf receptors (TfR; Aisen, 1998) where it is mainly used for heme biosynthesis and erythropoiesis (Andrews, 2000).

During pregnancy, maternal iron absorbance increases substantially throughout the gestational period [from 0.8 mg/day in the first trimester to 7.5 mg/day in the third trimester (Bothwell, 2000)] to fulfill the needs of the developing fetus. Increased iron absorption is also required for maternal erythropoiesis, to maintain hemoglobin (Hb) levels (Bothwell, 2000) and to increase the iron stores to compensate for blood loss during delivery (Milman, 2006). Studies have shown that 40–70% of iron that is transferred to the fetus derives from maternal iron stores (Murray and Stein, 1970; Bothwell, 2000), suggesting that fetus can mobilize iron from this resource; this will be further discussed.

Maternal iron status during pregnancy can be monitored by biomarkers such as Hb, hematocrit, serum ferritin (SF), serum soluble TfR (sTfR), and total body iron (TBI). SF concentration is a well established marker for iron reserves; SF levels ≤30 μg/L are indicative of low iron reserves and levels ≤12 μg/L are associated with iron deficiency (Milman, 2006). Soluble TfR are detached receptors from young erythrocytes which in high concentrations indicate iron deficiency at cellular level and may be useful marker to monitor erythropoiesis (Feelders et al., 1999). Both SF and sTfR measurements during pregnancy yield reliable information on maternal iron status; the sTfR-index (sTfR/log SF) is a practicable parameter for patients with depleted iron stores (Punnonen et al., 1997) and during pregnancy may correct for plasma volume expansion differences (Cao and O’Brien, 2013).

Another parameter for assessment of iron homeostasis is hepcidin, a hormone mainly produced by the liver that controls iron eﬄux. Hepcidin binds to ferroportin, induces internalization and degradation of this molecule, preventing iron export from the cell (Nemeth et al., 2004). Levels of hepcidin vary according to the iron concentrations in circulation, and kinetics of this hormone during pregnancy is indicative of the status of maternal iron stores (Simavli et al., 2014). In a cohort of healthy Northern European pregnant women, reduced levels of hepcidin correlated with decreased ferritin and iron concentrations, which occur simultaneously to an increase in sTfR-index in the third trimester (van Santen et al., 2013). As proposed in this study, low hepcidin production might reflect increased iron demands of the developing fetus and increase in maternal erythropoietic activity during pregnancy, which reduces maternal iron stores.

The effect of *Plasmodium* placental infection on maternal and cord blood iron parameters [hepcidin, iron, ferritin, sTfR, Tf saturation (TS)] has been considered. In a population from Gabon, anemic women with PM had a trend for lower levels of sTfR compared to anemic women without PM suggesting that parasitemia could affect proliferation of erythroid progenitors (Van Santen et al., 2011). A slight but not significant increase in ferritin concentrations was also observed in these anemic and infected pregnant women. Studies conducted in Malawi showed that infected pregnant women exhibited increased levels of ferritin compared to non-infected and that cord blood ferritin was correlated with increased maternal parasitemia and lower birth weight (Abrams et al., 2005). Moreover Hb levels in the fetus were not affected by infection suggesting that fetal iron stores are preserved. Other studies corroborate these observations showing that neither maternal or placental infection had an effect on Hb levels (Mokuolu et al., 2009; Van Santen et al., 2011) as well on iron parameters in the cord blood (Van Santen et al., 2011).

### IRON TRANSPORT FROM MOTHER TO FETUS

**FIGURE 1:**
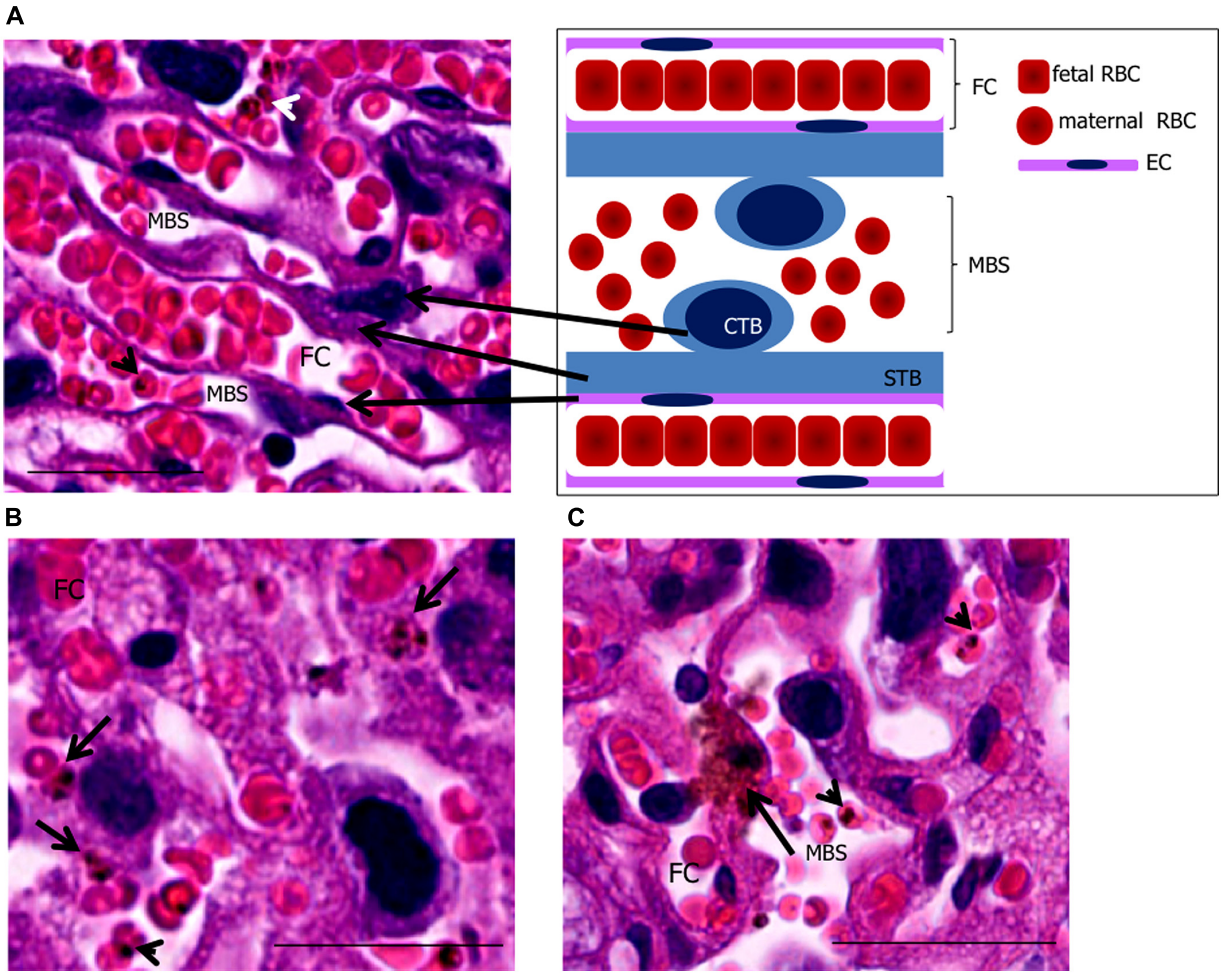
**Phagocytosis by trophoblasts of infected mouse placentas at end-stage gestation. (A)** placental histology showing trophoblasts [cytotrophoblasts (CTB) and syncythiotrophoblasts (STB)], maternal blood space (MBS), endothelial cell (EC), and fetal capillary (FC) corresponding to the scheme on the upper right-hand panel. **(B,C)** placental sections showing engulfed IEs (arrows in **B,C**) in trophoblasts. Arrowheads point to IEs. Scale bar 25 μm.

Both in mouse and human placentas, maternal blood is directly in contact with trophoblasts. These cells, more specifically named cytotrophoblast and syncythiotrophoblasts, are arranged in layers to form a barrier separating maternal from fetal blood and allow flux of gas and nutrient to the fetus through different mechanisms (Sibley et al., 2010; **Figure 1A**). Iron is transferred across the placenta via proteins involved in iron transfer; the detailed mechanisms regulating trophoblasts uptake of iron from maternal circulation and transfer to the fetus are still unclear. It has been suggested that iron uptake from maternal serum may occur primarily via DMT1 (Gruper et al., 2005). DMT1 is a transmembrane glycoprotein that transports divalent iron into the cytoplasm; it is located both in the cell membrane and on late endosomes (Andrews, 1999). Expression of DMT1 on the apical side of human trophoblasts (facing maternal blood) suggests DMT1 could play a major role in iron uptake by trophoblasts also during pregnancy (Gruper et al., 2005). In human term placentas, DMT1 is located predominantly on the maternal side of syncytiotrophoblasts, more rarely on the fetal side (Li et al., 2012b). Fetal iron uptake is also mediated by the expression of TfR on the apical membrane of human placenta (Kroos et al., 1996; Georgieff et al., 2000; Bastin et al., 2006). This suggests that the mechanism also operates to ensure iron transport from maternal circulation to the fetus. TfR is induced during pregnancy, in response to iron deficiency; it is rapidly transcytosed to the apical membrane of syncytiotrophoblasts (Gruper et al., 2005), as demonstrated in rodent (Gambling et al., 2001; Cornock et al., 2013) and human studies (Li et al., 2012a) of maternal iron deficiency.

Mechanisms of iron transfer from mother to fetus must be well *orchestrated* to guarantee adequate iron supply to the developing embryo. The cross-talk between mother, placenta (trophoblast) and fetus is not yet well understood but the *conductor* of the *symphony* seems to be the fetus. Studies have shown that fetal liver iron status directly correlates with TfR expression on placenta and maternal liver, suggesting that the embryo regulates iron uptake by the mother and at the trophoblast level (Gambling et al., 2009). In a rat model of dietary iron deficiency using two different strains, placental TfR and DMT1 expression were upregulated, possibly to increase iron transport and supply to the fetus (Cornock et al., 2013). These studies also showed inter-strain differences in maternal liver iron contents in controls not associated to expression of duodenal iron transporters but rather placental efficiency in iron uptake and transfer to the embryo. In the placenta TfR seems to be modulated by hepcidin; studies on hepcidin transgenic embryos have shown that expression of TfR mRNA was significantly downregulated in the placentas, suggesting that hepcidin is constitutively produced by the fetal liver and possibly controlled by fetal iron contents (Gambling et al., 2009) modulating the iron uptake by trophoblast (Martin et al., 2004). Underlying mechanisms are still unclear. Taken together these data strongly suggest that fetal iron status regulates trophoblast iron uptake.

Much evidence supports ferroportin 1 (FPN1) as the major iron export molecule due to its high expression on the basal membrane of the syncytiotrophoblast (Donovan et al., 2000; McKie et al., 2000; Bastin et al., 2006). However, the observation that FPN mRNA expression (Gambling et al., 2001; Li et al., 2008) and protein levels (Gambling et al., 2009; Cornock et al., 2013) on trophoblasts of rat placentas were not altered regardless of maternal iron status suggests that regulation of iron eﬄux from trophoblast may not involve the FPN-hepcidin pathway (Gambling et al., 2009; Cornock et al., 2013). The observation that DMT1 is also localized on the syncytiotrophoblast basal membrane raises the possibility that this molecule could be involved in iron eﬄux to the fetal serum (Georgieff et al., 2000).

### IRON DEFICIENCY AND PREGNANCY MALARIA

In malaria endemic regions, pregnant women with nutritional iron deficiency who are infected with *Plasmodium* may be protected from PM (Kabyemela et al., 2008) but are at risk of developing severe anemia (Guidotti, 2000) increasing the risk of perinatal mortality and morbidity (Levy et al., 2005). The apparent contradiction stems from two distinct effects of iron: low levels may impair fetal development; high concentration could promote parasite replication, as this metal is strictly required for the viability of these pathogens (Cabantchik et al., 1996; Ganz, 2009). Iron supplementation trials have shown contradictory results on pregnancy outcomes during malaria infection (Nacher et al., 2003; Friedman et al., 2009). Iron and acid folic supplementation has been recommended for all women of child-bearing age in malaria endemic regions with the aim of overcoming iron deficiency. Safety of this strategy in terms of malaria risk has not been assessed and remains a concern. Increased malaria risk has been observed in pregnant women who receive intravenous iron (Oppenheimer et al., 1986) but oral iron supplementation increased neither the prevalence nor severity of malaria in Gambian women (Menendez et al., 1994). In contrast, iron deficiency has been associated with decreased susceptibility to *P. falciparum* infection in pregnant women in Tanzania (Kabyemela et al., 2008) but PM appeared to protect Malawian pregnant women from maternal iron deficiency (Senga et al., 2011). Likewise Sudanese women with *P. falciparum* PM had low frequency of anemia (Adam et al., 2012). These epidemiological findings suggest impairment of iron metabolism over the course of malaria infection during pregnancy. On the one hand, decreased iron availability appears to protect against PM, probably by depriving the parasite of iron required for expansion during blood stage infection. On the other hand, PM appears to protect pregnant women from developing iron deficiency, possibly by impairing the mechanism of iron transport in the placenta. Dysregulation of iron metabolism and distribution in malaria infection may be exacerbated during pregnancy, due to increased maternal erythropoiesis and fetal needs. There is little information on whether alterations in the intricate interaction between maternal and fetal iron metabolism in PM impose restrictions on fetal growth and survival. There are so far no hypotheses to explain the interplay between iron homoeostasis, pregnancy status, and malaria infection.

## IRON DYSREGULATION IN THE PM MODEL

### TROPHOBLAST RESPONSE TO INFECTION

**FIGURE 2:**
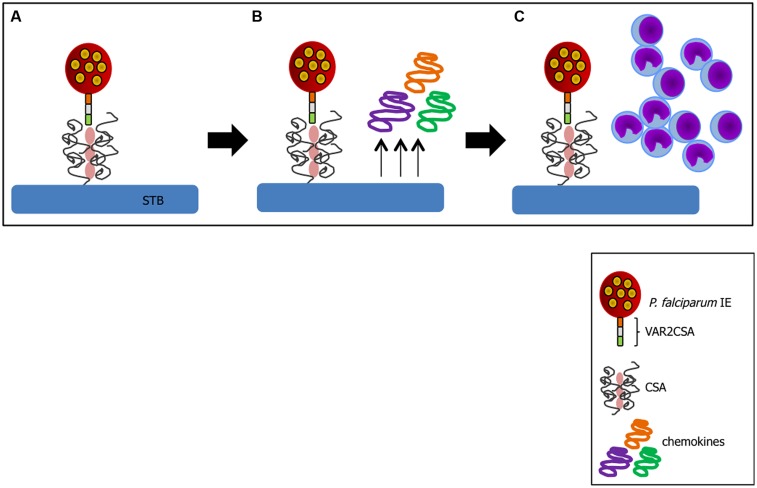
**Trophoblast response to IE binding. (A)** Cytoadhesion of IE to trophoblast (TB) via VAR2CSA-CSA interaction leads to chemokine production by these activated cells **(B)** and recruitment of inflammatory cells to the site **(C)**. Sequence of events compiled based on studies from (Fried and Duffy, 1996; Salanti et al., 2004; Lucchi et al., 2008; Srivastava et al., 2010).

A critical factor in PM pathogenesis is sequestration of IEs in the placenta. This process may occur through interaction of VAR2CSA exposed on IE membrane with CSA expressed on trophoblasts (Fried and Duffy, 1996) or by arrest of IEs in maternal low blood flow areas (de Moraes et al., 2013). In *P. falciparum* infection, VAR2CSA is so far the best characterized molecule involved in cytoadherence (Salanti et al., 2003, 2004) and a favored target of PM vaccines (Kane and Taylor-Robinson, 2011). Trophoblasts can respond to infection after IE binding. *In vitro* studies with *P. falciparum* have shown that following IE-trophoblast interaction placental cells produce chemokines such as macrophage inflammatory protein-1 alpha (MIP-1α/CCL3) and macrophage migration inhibitory factor (MIF) via activation of c-Jun N-terminal kinase I (JNK I) which in turn recruit peripheral blood mononuclear cells to the site (Lucchi et al., 2008); this inflammatory milieu contributes to PM pathology (**Figure 2**). Trophoblasts have also been shown to respond to *Plasmodium* products such as hemozoin (crystallized heme from Hb digestion during the blood stage of *Plasmodium* infection; Lucchi et al., 2011), *P. chabaudi* IEs (Poovassery and Moore, 2009), and pathogenic bacteria (Griesinger et al., 2001) releasing pro-inflammatory factors.

Despite VAR2CSA-CSA interactions being exclusive to *P. falciparum*-induced PM, the observation that other *Plasmodium* parasites are capable of causing malaria during pregnancy both in humans (Poespoprodjo et al., 2008) and mice (Neres et al., 2008; Rodrigues-Duarte et al., 2012) suggests involvement of other parasite molecules that could target CSA. This is supported by *in vitro* data showing that *P. berghei* IE binding is CSA-dependent (Neres et al., 2008). The contribution of VAR2CSA and non-VAR2CSA molecules to PM is currently being addressed by the characterization of a PM mouse model induced by transgenic *P. berghei* parasite that expresses VAR2CSA molecule (de Moraes et al., unpublished data). Our recent study of blood circulation patterns in mouse placenta using intra-vital microscopy showed that IEs and non-IEs are arrested in regions where maternal circulation is stationary (de Moraes et al., 2013) suggesting that here *P. berghei* IE sequestration might be independent of molecular interactions with trophoblasts.

Stationary IEs in low maternal blood spaces can be engulfed by trophoblasts. Phagocytic activity of trophoblasts occurs in early stages of pregnancy, during implantation of blastocyst (Tachi et al., 1970; Bevilacqua and Abrahamsohn, 1989); engulfment of maternal components such as endometrial cells, red blood cells, and epithelial cells creates space for embryo attachment to the endometrium and its nutrition (Bevilacqua and Abrahamsohn, 1989). After maturation, trophoblasts seem to retain their phagocytic activity. Studies have shown that trophoblasts from mid-stage gestation are capable of phagocytosing erythrocytes (Albieri and Bevilacqua, 1996), *P. chabaudi* IEs (Poovassery and Moore, 2009), *P. berghei* parasitic material *in vitro* (Pavia and Niederbuhl, 1991) as well as yeast and bacteria *in vivo* (Amarante-Paffaro et al., 2004). Our recent observation using intra-vital microscopy show that in placentas from gestational day 18, *P. berghei* IEs are engulfed by trophoblasts supporting the idea that phagocytic function is extended until termination of pregnancy (de Moraes et al., 2013; **Figures 1B,C**).

### DISRUPTION OF INTRACELLULAR HEME HOMEOSTASIS AS A MECHANISM INVOLVED IN EXCESSIVE IRON ACCUMULATION IN TROPHOBLAST

**FIGURE 3:**
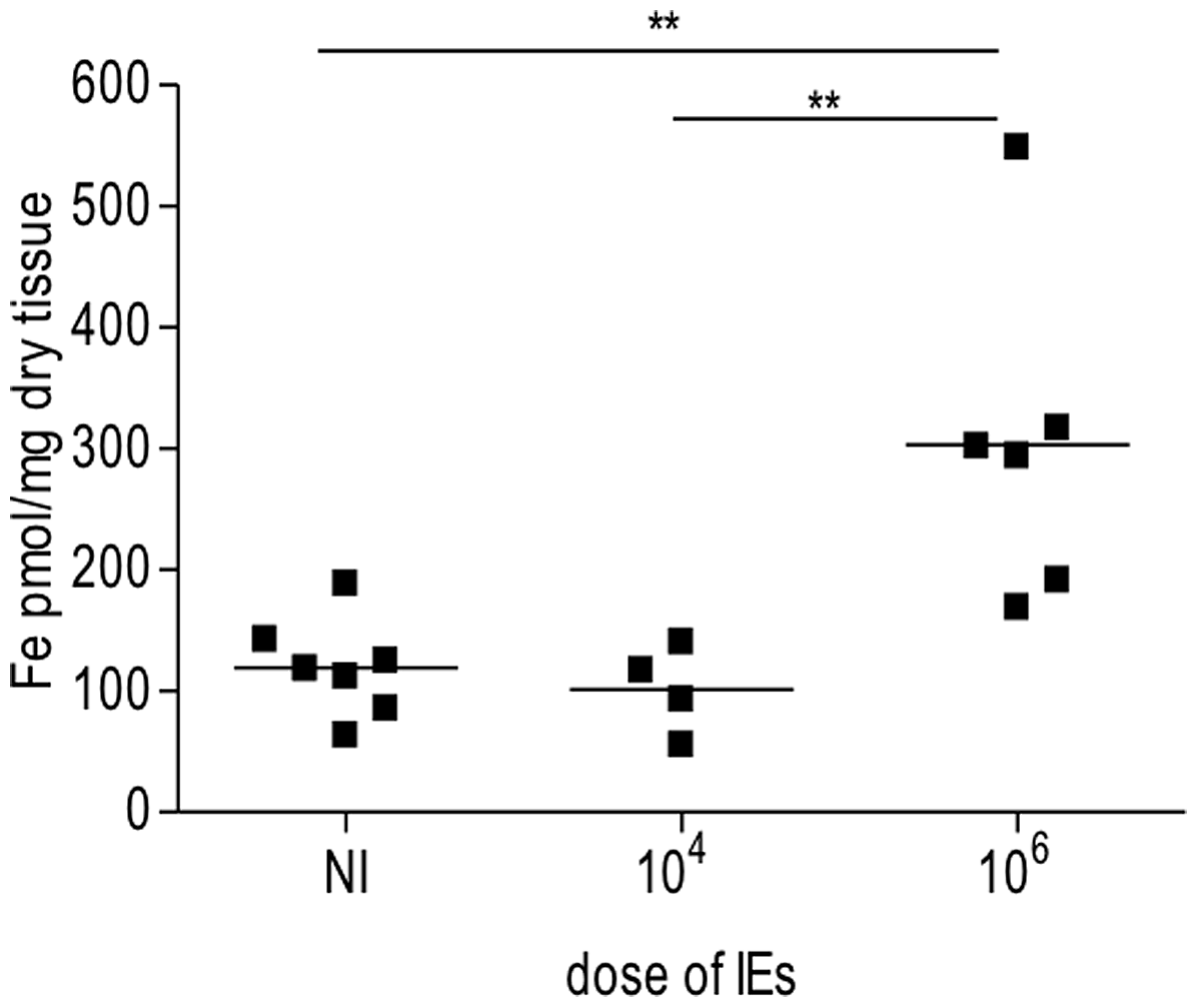
**Iron accumulation in placenta is dependent on dose of infection.** Pregnant mice were infected with either 10^4^ or 10^6^ *Plasmodium berghei*-infected erythrocytes (IEs). Placentas from *Plasmodium*-infected pregnant mice were collected at G19/20 and processed for iron measurements as previously described (Gozzelino et al., 2012). Briefly placentas were dehydrated overnight, dissolved in 3 M HCl/trichloroacetic acid (TCA) 10% for 24 h. Labile iron was detected by colorimetric reagent (bathophenanthroline-disulfonic acid; BPTS) and absorbance was measured by spectrophotometer (SmartSpec 3000, Bio-Rad); **p < 0.01.

In our PM model, abortions, stillbirths, underweight babies, and maternal mortality are consequences of *Plasmodium* infection during pregnancy. These features are associated with irreversible placental damage, due to IE accumulation. Here we present a novel hypothesis as to the mechanisms underlying fetal death in PM. Fetal death/loss during mid-gestation (abortions) has already been shown to be associated to pro-inflammatory cytokine response to *Plasmodium* infection in the mouse (Poovassery and Moore, 2009; Poovassery et al., 2009) and in humans (Okoko et al., 2003), and to microcirculatory impairments (Avery et al., 2012). In our model fetal death was observed at end-stage gestation (G19/20): 8/30 fetuses were expelled at G19 and were very small for gestational age. The remaining fetuses were assessed *in utero*, were in general fully developed but underweight. Our preliminary data show iron accumulation in trophoblasts in 43% of the infected placentas from dead fetuses (**Table 1**), which was associated to the dose/number of *Plasmodium*-IEs used in infection of pregnant mice (**Figure 3** and **Table 2**).

**Table 1:** Iron accumulation in placentas from live and dead fetuses.

Iron accumulation	Live fetuses (*n* = 20)	Dead fetuses (*n* = 30)
+	0	13
-	20	17

*Association between phenotypes (iron accumulation and death): *p* < 0.01 (chisquare test; df = 1).*

**Table 2:** Iron accumulation in placentas from dead fetuses according to IE dose.

Iron accumulation	10^6^ IEs (*n* = 15)	<10^6^ IEs (*n* = 14)
+	13	0
-	2	14

*Association between phenotypes (iron accumulation and IE dose): *p* < 0.01 (chisquare test; df = 1).*

**FIGURE 4:**
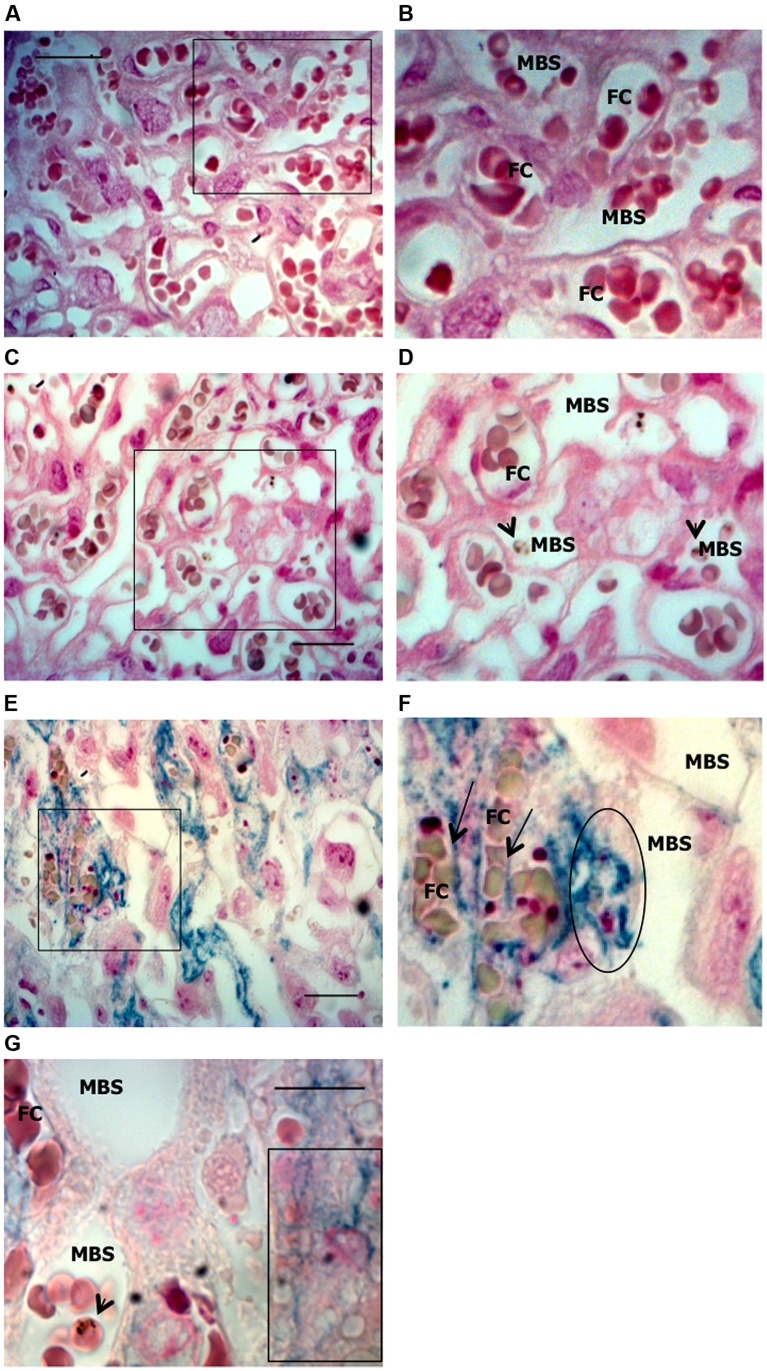
**Iron accumulation in trophoblasts.** Placentas from *Plasmodium*-infected pregnant females were evaluated at G19 for iron accumulation by Prussian blue staining in placental sections. **(A,B)** Non-infected placenta showing maternal blood space (MBS) and fetal capillary (FC) separated by trophoblast. **(C,D)** Infected placenta from a live fetus that shows no iron accumulation in trophoblasts and fairly preserved tissue structure; **(E,F)** Infected placenta from a dead fetus showing significant iron accumulation (blue) in trophoblast and loss of integrity of the tissue structure. Iron seems to be accumulated in phagocytic trophoblasts (surrounded area in **F** and box in **G**) and at the interface between trophoblast and fetal capillary (arrows in **F**) but not in MBS **(E–G)**. Areas delimitated in **A,C,E** are magnified (crop and zoom) in **B,D,F,** respectively. Scale bars: 25 μm **(A,C,E)** and 10 μm **(G)**. Arrowheads point to infected erythrocytes.

Iron deposits were assessed in placental sections by Perl’s Prussian blue staining (**Figure 4**). The pattern of excessive iron staining in placentas from dead fetuses (**Figures 4E,F**) was observed neither in non-infected (**Figures 4A,B**) nor in infected placentas from live but underweight fetuses (**Figures 4C,D**); this strongly suggests that iron overload in trophoblasts is associated with the most severe pathological events. Iron was accumulated inside trophoblasts (**Figures 4E,F**) and, in particular, at the interface between trophoblasts and fetal blood capillary and, possibly in fetal circulation (**Figure 4F**). Although further experiments would be required to validate the latter observation, staining was not observed in maternal blood spaces (**Figures 4E–G**) supporting the specificity of the results obtained. We also observed loss of placental tissue integrity and cellular damage in regions of iron positive staining (**Figure 4E**), which suggests that iron overload in trophoblasts might induce programmed cell death, presumably mediated by oxidative stress. Increased apoptosis of trophoblasts has been linked to adverse pregnancy outcomes such as low birth weight due to intrauterine growth retardation (IUGR; Endo et al., 2005), spontaneous abortion (Olivares et al., 2002; Nakashima et al., 2008), and in a mouse model of intrauterine fetal death (Mu et al., 2003). In an experimental model of iron overload during pregnancy, iron accumulation in trophoblasts showed a fivefold increase but fetal iron content was not modified, suggesting that transfer of iron across the placenta is, to quote, *a rate-liming step* (Martin et al., 2004). Despite these evidences, we would not rule out the possibility that, in our model, excessive iron in the placenta is toxic for the fetus; the observation that iron accumulates at the interface with fetal capillaries and possibly in the fetal circulation allows us to speculate toward this direction.

The mechanism proposed to explain iron accumulation in our model relies on dysregulation of intracellular heme homeostasis leading to iron overload presumably occurring as a consequence of increased IE phagocytosis by trophoblasts. These cells are capable of engulfing and digesting red blood cells (Albieri and Bevilacqua, 1996) possibly for fetal nutrition at the beginning of gestation (Bevilacqua and Abrahamsohn, 1989); hence, it is possible that the mechanisms involved in iron recycling from Hb in the trophoblast occurs as in macrophages (Bratosin et al., 1998). We would expect that following phagocytosis, disrupted erythrocytes would lead to intracellular accumulation of cell-free Hb, a tetramer responsible for oxygen transport (Bratosin et al., 1998) which upon oxidation releases its heme prosthetic groups (Schechter, 2008). Non-Hb bound heme (free heme) sensitizes non-hematopoietic cells to undergo programmed cell death in response to pro-inflammatory agonists such as tumor necrosis factor (TNF) released in the course of the infection (Seixas et al., 2009). This deleterious effect is caused by the iron atom contained within the protoporphyrin ring of the heme molecule and its ability to produce highly reactive hydroxyl radicals via its participation in the Fenton chemistry (Gozzelino et al., 2012).

Degradation of heme by the heme catabolizing enzyme heme oxygenase 1 (HO-1) prevents heme-induced iron cytotoxicity and generates equimolar amounts of labile iron (Fe^2+^), carbon monoxide (CO), and biliverdin (Tenhunen et al., 1968; Choi and Alam, 1996; Soares and Bach, 2009; Gozzelino et al., 2010). Expression of HO-1 is induced in a variety of stress responses, including to heme itself and is encoded by *HMOX1* gene (Tenhunen et al., 1968). This is achieved by suppressing the activity of Bach-1, transcriptional repressor of *Hmox-1* gene (Sun et al., 2002; Gozzelino et al., 2010). The crucial role of HO-1 has already been demonstrated in cerebral and severe forms of malaria (Pamplona et al., 2007; Seixas et al., 2009; Ferreira et al., 2011); increased expression of this detoxifying enzyme strongly correlates with the ability to survive the infection. The protection afforded by HO-1 relies on the inhibition of heme sensitization to programmed cell death.

To limit pro-oxidant effects of iron, a regulatory mechanism evolved to couple heme catabolism and maintenance of iron homeostasis. This metabolic iron adaptation is conferred by the expression of ferritin heavy/heart chain (FtH; Gozzelino and Soares, 2014). Conversion of Fe^2+^ into inert Fe^3+^ is attributed to the ferroxidase activity of FtH, a property necessary to neutralize the redox activity of labile iron and prevent generation of oxidative stress (Eisenstein et al., 1991; Harrison and Arosio, 1996). This allows storage of iron inside the multimeric complex of ferritin, formed by 24 subunits of FtH and ferritin light/liver chain (FtL; Arosio and Levi, 2002) at proportions that varies between different tissues (Harrison and Arosio, 1996). The iron storage capacity of the multimeric complex of ferritin can incorporate up to 4500 iron atoms (Harrison and Arosio, 1996), as inorganic ferrihydrite aggregates from which are then released according to cellular requirements (Koorts and Viljoen, 2007). The mechanism of release is currently not understood.

The cytoprotective effect of FtH relies on its anti-oxidant properties, which reduce free radicals production (Balla et al., 1992). Reactive oxygen species (ROS) are responsible for sustained activation of JNK (Kamata et al., 2005) which induces caspase-3 cleavage and programmed cell death (Gozzelino et al., 2012). Through inhibition of ROS, FtH diminishes JNK phosphorylation (Pham et al., 2004) preventing cell death. In malaria infection, FtH was shown to prevent tissue damage and reduces disease severity by providing a metabolic adaptation to tissue iron overload; this enables mice and humans to survive to the infection (Gozzelino et al., 2012). Further experiments would be required in our PM model to assess the mechanisms by which FtH can protect against trophoblast damage.

Intracellular accumulation of free heme is also controlled by the expression of proteins that regulate heme trafficking inside the cells, known as heme transporters (Yuan et al., 2013). Extracellular release of excessive heme is mediated by the expression of membrane proteins that act as heme exporters, such as BCRP/ABCG2 (Krishnamurthy and Schuetz, 2005) and two isoforms of the FLVCR1 (Keel et al., 2008; Chiabrando et al., 2012). FLVCR1a is a cell surface molecule and regulates heme extracellular exit (Keel et al., 2008; Vinchi et al., 2014); FLVCR1b is a mitochondrial protein that controls heme eﬄux into the cytosol for hemoglobinization and erythroid differentiation (Chiabrando et al., 2012).

**FIGURE 5:**
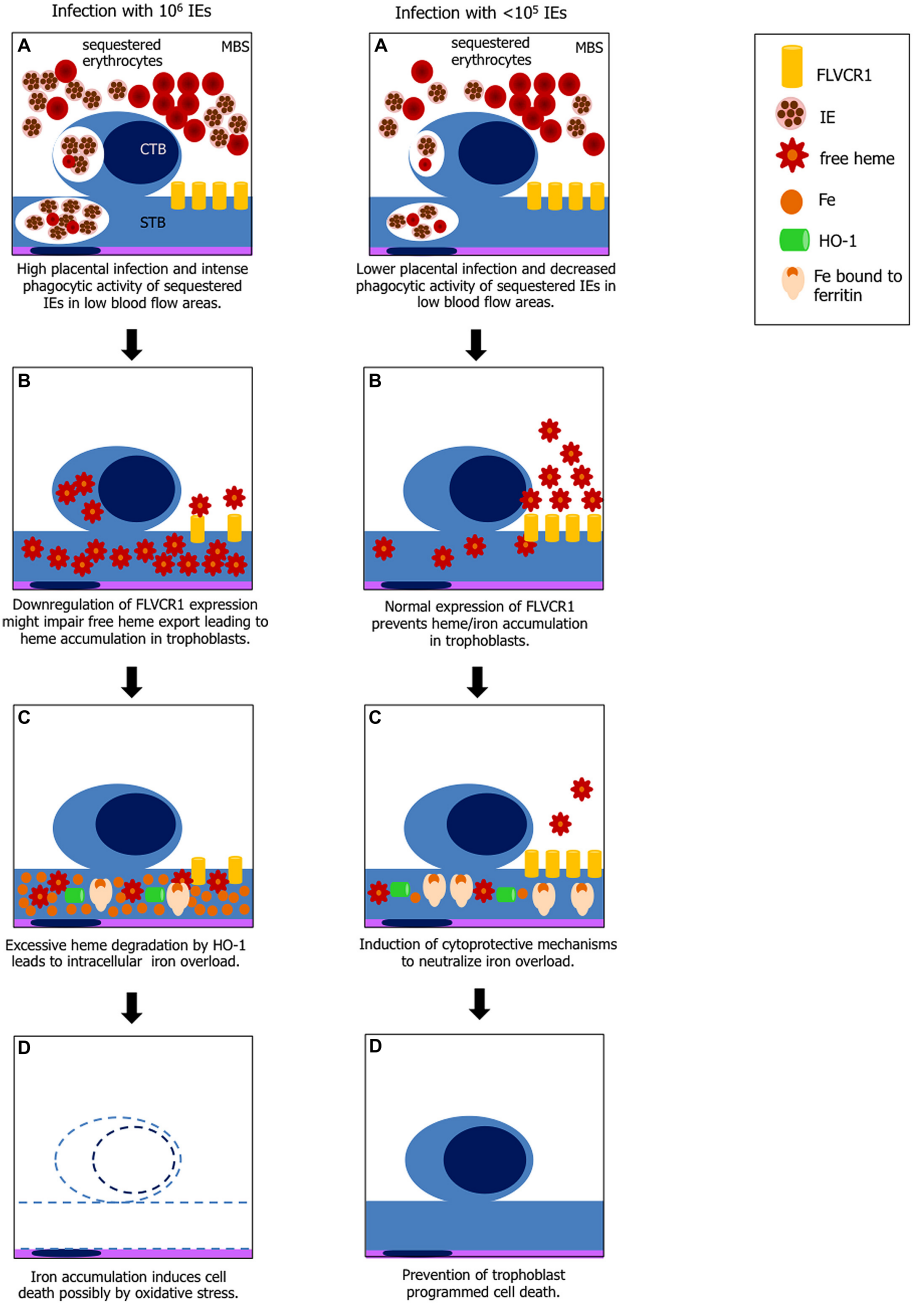
**Hypothesis explaining heme-iron overload in trophoblast according to IE load.** Left panel: **(A)** increased phagocytosis of IEs and possibly non-IEs by cytotrophoblast (CTB) and syncythiotrophoblasts (STB) in low blood flow regions in maternal blood space (MBS) where IE and non-IE are sequestered; **(B)** disruption of erythrocytes in the phagocytic vacuole leads to free heme exposure; downregulation of FLVCR1a on trophoblasts might cause free heme accumulation due to decreased heme export; **(C)** heme catabolism by HO-1 generates large amounts of iron which may exceed ferritin binding capacity; **(D)** free iron (Fe^2+^) induces cell death by ROS. Right panel: **(A)** lower placental infection might not affect FLVCR1a expression and phagocytosis is decreased; **(B)** excessive free heme is exported from the cell; **(C)** iron from heme catabolism is neutralized by ferritin; and **(D)** trophoblasts are protected from programmed cell death.

Our preliminary data also show that FLVCR1a mRNA expression in infected placentas is downregulated (data not shown) suggesting that reduced FLVCR1a expression together with intense trophoblast phagocytic activity may contribute to intracellular heme accumulation (**Figure 5**). Placenta has been shown to express high amounts of FLVCR1 (Jaacks et al., 2011) but the functional roles of this molecule in this tissue are poorly investigated. FLVCR1 is required for erythroid differentiation maintaining the balance of intracellular free heme during erythropoiesis (Quigley et al., 2004) and has an important role in iron recycling by macrophages (Keel et al., 2008). It has been suggested that expression of FLVCR1 in the placenta may reverse the flow of heme from this tissue to maternal circulation, so to prevent the fetoplacental unit from iron toxicity (Cao and O’Brien, 2013). Analysis of FLVCR1 expression in a population of pregnant adolescents at high risk of iron deficiency showed that FLVCR1 was downregulated in placentas of anemic mothers at term (Jaacks et al., 2011). In our PM model it is possible that placental FLVCR1a downregulation may be linked to maternal anemia induced by *Plasmodium* infection. Engulfing high numbers of sequestered IEs in low maternal blood flow regions by trophoblasts expressing low levels of FLVCR1a could result in heme accumulation. We have not yet determined if Hb levels and FLVCR1a expression correlates to IE expansion in the placenta, which could possibly explain iron overload in placentas from mice infected with higher parasite numbers.

We could argue that mechanisms involved in the putative heme-induced cellular damage observed in infected placentas could be associated with a decrease in expression of cytoprotective proteins, such as HO-1 and ferritin. These proteins cooperate to degrade heme and neutralize the iron released from the protoporphyrin ring of the heme molecule (Gozzelino and Soares, 2014). Whilst HO-1 is responsible for heme catabolism, expression of ferritin is crucial to neutralize iron generated through heme degradation and store it inside its multimeric structure (Gozzelino and Soares, 2014). This would lead to insufficient function to prevent the damage induced by intracellular heme accumulation. In fact, administration of anti-oxidant compounds that mimic the cytoprotective effect of HO-1 prevented heme-mediated cytotoxicity and pathological outcomes of *Plasmodium* infection in mice (Seixas et al., 2009). However, a recent study in mice with conditional disruption of *Flvcr1a* gene in the liver showed that in the absence of FLVCR1a heme and iron accumulated in this organ and that genes such as *Ho-1, Fpn, H-Ft* and *L-Ft* were upregulated suggesting that FLVCR1a is associated to heme degradation (Vinchi et al., 2014). Therefore it is possible that in our model heme degradation pathway in trophoblasts is upregulated to compensate for the impaired function of heme export by FLVCR1a, as previously suggested (Vinchi et al., 2014).

Heme is synthesized in the mitochondria by a series of enzymatic reactions and exported to the cytosol to be incorporated in hemoproteins (reviewed in Chiabrando et al., 2014). Mice lacking FLVCR1a in the liver also showed downregulation of genes involved in the heme biosynthesis after injection of heme precursor 5-aminolevulinic acid (ALA) whereas in controls treatment induced FLVCR1a upregulation (Vinchi et al., 2014). Extrapolation of this evidence to our model allow the speculation that free heme accumulation after intense phagocytosis of IEs by trophoblast could repress heme *de novo* biosynthesis which in turn downregulates FLVCR1a expression.

Whether there is a role for HO-1 in protection against PM remains to be established. The notion that this protein is essential for processes such as oocyte maturation, fertilization, fetus implantation, and acceptance, Bainbridge and Smith (2005) and Zenclussen et al. (2007) suggests that the positive potential of HO-1 in PM is worth to be tested.

## FINAL REMARKS

Our PM model represents a severe form of the disease; mechanisms underlying fetal death are still unclear. Our initial observation that only 43% of placentas from dead fetuses showed iron accumulation in trophoblasts (**Table 1**) suggests that iron overload in PM is a heterogeneous and progressive process and potentially represents one of the mechanisms to explain fetal demise. The amount of iron in the placenta was significantly associated with infection dose (**Figure 2** and **Table 2**) implying that the higher the degree of infection the higher the probability of iron accumulation. Pregnancy outcomes are improved when mice are infected with IE doses 100× times lower than usual; incidence of fetal death is greatly decreased and birth weight is comparable to normal (unpublished) suggesting that here infection does not have an important impact on fetal development. It could also indicate that lower infection levels may not deprive the organism of the capacity to activate cytoprotective molecules controlling regulation of heme/iron homeostasis. This is further supported by the observation that iron accumulation in infected placentas is associated with the number of injected IEs.

The main hypothesis we formulate in this article is that increased phagocytosis of IEs by trophoblasts leads to intracellular iron overload as a consequence of accumulation of free heme after IE disruption. Based on preliminary evidences we suggest that infection induces downregulation of FLVCR1a which affects heme homeostasis triggering cytotoxic events in trophoblasts and impairing fetal development.

Whether *Plasmodium* infection induces dysregulation of molecules involved in heme-iron uptake, storage and/or eﬄux leading to intracellular iron overload is highly speculative. Nevertheless, our observations that iron was accumulated at the interface between trophoblast basal membrane and fetal capillary encourage the investigation of FPN expression levels in the infected placenta. We could further elaborate that insufficient function of FLVCR1a in exporting intracellular heme together with inefficient iron export through FPN (if shown to be downregulated) might both contribute to iron accumulation in trophoblasts. Our current observations warrant further efforts to investigate iron metabolism in PM.

## Conflict of Interest Statement

The authors declare that the research was conducted in the absence of any commercial or financial relationships that could be construed as a potential conflict of interest.
